# Relationship between maternal employment status and children’s food intake in Japan

**DOI:** 10.1186/s12199-021-01026-z

**Published:** 2021-10-28

**Authors:** Sachie Mori, Keiko Asakura, Satoshi Sasaki, Yuji Nishiwaki

**Affiliations:** 1 Department of Environmental and Occupational Health, Toho University Graduate School of Medicine, Tokyo, Japan; 2 Department of Environmental and Occupational Health, School of Medicine, Toho University, Omori-Nishi 5-21-16, Ota-ku, Tokyo, 143-8540 Japan; 3 Department of Social and Preventive Epidemiology, School of Public Health, The University of Tokyo, Tokyo, Japan

**Keywords:** Maternal employment, Food intake, Primary school children, Nutrition knowledge, Dietary attitude, Family environment

## Abstract

**Background:**

Although long maternal working hours are reported to have a negative effect on children’s dietary habits, few studies have investigated this issue in Japan. Healthy dietary habits in childhood are important because they may reduce the risk of future disease. Here, we examined the relationship between maternal employment status and children’s dietary intake in 1693 pairs of Japanese primary school 5th and 6th graders and their mothers.

**Methods:**

The survey was conducted using two questionnaires, a brief-type self-administered diet history questionnaire and a lifestyle questionnaire. The analysis also considered mothers’ and children’s nutrition knowledge, attitudes toward diet, and some aspects of family environment.

**Results:**

Longer maternal working hours were associated with children’s higher intake of white rice (g/1000kcal) (*β* 11.4, 95%CI [1.0, 21.9]; working ≥8h vs. not working), lower intake of confectioneries (g/1000kcal) (*β* −4.0 [−7.6, −0.4]), and higher body mass index (BMI) (kg/m^2^) (*β* 0.62 [0.2, 1.0]). Although maternal employment status was not significantly associated with lower intake of healthy food (e.g., vegetables) or higher intake of unhealthy food (e.g., sweetened beverages) in the children, in contrast with previous studies, it may have affected children’s energy intake through their higher intake of white rice. Further, children’s nutrition knowledge and attitudes toward diet, mothers’ food intake, and some family environment factors were significantly associated with intakes of vegetables and sweetened beverages in the children.

**Conclusions:**

Longer maternal working hours were significantly associated with higher intake of white rice and lower intake of confectioneries, as well as higher BMI among children. Even when a mother works, however, it may be possible to improve her child’s dietary intake by other means such as nutrition education for children or enhancement of food environment.

## Background

In Japan, care for children at home is usually provided by the mother. Even when the wife works full time, the reported percentage of husbands in charge of meal preparation more than once or twice a week is only 32.5%. This rate decreases to 21.6% if the mother works part time, 15.9% if she is self-employed, and 18.1% if she is a housewife [1]. Three-generation cohabitation—which allows more sharing of cooking and housework between mothers and other family members—was previously relatively frequent in Japan, but the rate has declined over recent decades, to only 5.3% of all households surveyed in 2018 [2].

Many studies have reported that long working hours by mothers have negative effects on their children’s dietary habits, including a lower frequency of breakfast intake [3], increased intake of unhealthy foods (e.g., processed foods high in fat and sugar [4], decreased intake of vegetables and fruits [5], and poor diet quality [3, 4, 6, 7]. Most of these studies were conducted in Western countries; however, the importance of examining the relationship between maternal employment and the dietary intake of children is emphasized by findings that dietary habits in childhood affect those in adulthood [8] and that good eating habits in childhood may reduce the future risk of noncommunicable diseases [9, 10]. This topic is particularly important in Japan given that the employment rate of Japanese mothers with a youngest child aged 9–11 years rises every year [2]. Of particular interest, the better socioeconomic status provided by a mother’s income may allow an improvement in the quality of the family diet; but at the same time—if an undesirable relationship does exist—it is also necessary to identify factors that might improve the diet of affected children.

In this study, we investigated the relationship between maternal employment status and children’s food intake in Japanese primary school children in the 5th and 6th grade (i.e., age 10 to 12 years). We also considered mothers’ and children’s nutrition knowledge, attitude toward diet, and some family environment factors as possible confounders of the relationship to clarify mutual associations among these factors.

## Methods

### Participants

The survey was conducted in 14 public primary schools in a prefecture in the Kanto area, the central part of the main island of Japan, in May 2018. Seven cities and towns were chosen from five administrative districts in the prefecture, based on survey feasibility. Two public primary schools with similar characteristics (e.g., number of enrolled children, location (urban/rural)) were then selected from each city/town by the municipal boards of education. These 14 primary schools enrolled 2650 children as 5th and 6th graders in April 2018, all of whom were recruited into the study. At the same time, we also recruited the children’s guardians, most of whom were the main preparers of their meals. No exclusion criteria were set, because all children attending public schools in Japan are required to be educated equally.

### Questionnaires and variables

Children and their guardians were surveyed using two questionnaires each. The first was a lifestyle questionnaire that asked about basic characteristics and nutrition knowledge, as well as attitudes and behaviors toward diet, and the second was a brief-type self-administered diet history questionnaire (BDHQ; BDHQ15y for children and BDHQ for adults) that assessed dietary intake.

The main exposure was whether or not the mother was working, and the number of working hours per day among those who were. The mothers were asked to answer the following question in the lifestyle questionnaire: “How many hours do you work a day? If you are not working, select ‘not currently working’”. Working was divided into three categories: (1) not working, (2) less than 8 h a day (<8), and (3) 8 or more hours a day (≥8). The cut-off point was based on the fact that the legal number of working hours in Japan is 8 h [11].

The outcome was children’s food intake, as estimated using the BDHQ15y, an instrument for primary, junior high, and high school students. It was developed from the BDHQ, which was designed to quantify food and nutrient intakes in Japanese adults over the preceding month through 80 questions that calculate the intake of 58 foods and over 100 nutrients. A dedicated calculation program is used to calculate food and nutrient intake. Food intake obtained from the BDHQ has been validated using food intake from semi-weighed dietary records [12]. In addition, nutrient intake obtained from the BDHQ has been validated [13]. The children responded to the BDHQ15y at home with their guardians. In the present analysis, food intake was energy-adjusted by the density method [14] and expressed as food intake per 1000 kcal of energy intake (g) [15].

The information below was collected using the lifestyle questionnaire. The children answered questions about sex, grade, nutrition knowledge, attitude toward diet, frequency of eating out for dinner, and the frequency of communication about diet with their guardians. The mothers answered questions about age, employment status and working hours, nutrition knowledge, attitude toward diet, sleeping hours, socioeconomic status, cohabitants (family structure), and the frequency of communication about diet with their child.

The nutrition knowledge questionnaire used to measure the nutrition knowledge of the children and their mothers has been validated [16]. This questionnaire includes the following sections: (1) knowledge about foods as nutrient sources, (2) physiological functions of nutrients in the body, (3) awareness of dietary recommendations (only for adults), and (4) relationship between nutrients and health outcomes. The percentage (%) of correct answers was calculated. Attitude toward diet was defined as follows. The children were asked “Do you pay attention to whether your diet is healthy or not?”, and given one answer choice (always, often, sometimes, or rarely). The children’s attitude toward diet was regarded as “adequate” for those who chose “always” or “often”, and “inadequate” for those who chose “sometimes” or “rarely.” The mothers were asked to respond to six statements: (1) I am careful to eat a balanced diet, (2) I am careful not to overeat, (3) I am careful to eat more fruits and vegetables, (4) I am careful to eat low-fat foods, (5) I am careful to avoid salty foods, and (6) I am careful to avoid foods with a high sugar content. They selected one response (totally agree, agree, not so much, or disagree) to each statement, which were scored as 3 for “totally agree,” 2 for “agree,” 1 for “not so much,” and 0 for “disagree.” These scores were then summed, giving a possible total range for attitude of 0 to 18. Two groups of equal size were then established by dividing mothers into 0–10 and 11–18 score groups, which were considered to represent “inadequate” and “adequate” attitudes, respectively. The mothers were also asked about their subjective socioeconomic status (SES), with answers selected from the five choices of “very straitened,” “straitened,” “average,” “affluent,” or “very affluent.” These were then further categorized as “straitened” for those who chose “very straitened” or “straitened,” “average,” and “affluent” for those who chose “affluent” or “very affluent.” The children were asked about the frequency of eating out for dinner, with responses from the four choices of “twice a week or more,” “between once a week and once a month,” “less than once a month,” or “almost never.” The children and mothers were both asked about the frequency of communication about diet, as follows: “Do you discuss meals, food, nutrition, etc. with your guardian?” (or “your child” in the statements for mothers), with responses from the four choices of “often,” “sometimes,” “not often,” or “rarely.” These were then further categorized as “high” for “often” and “sometimes,” and “low” for “not often” and “rarely.” The state of communication was then classified into four groups based on the combination of “high” and “low” answers by the children and their mothers (child (C):low-mother (M):low; C:low-M:high; C:high-M:low; C:high-M:high) and treated as a categorical variable.

### Measurements

Body height and weight were measured to the nearest 0.1cm and 0.1 kg, respectively, with the child wearing light clothing and no shoes. Measurement was done as part of a routine health check-up by school nurses at each school from April to June in 2018. Body mass index (BMI) was calculated as body weight divided by the square of body height (kg/m^2^).

### Statistical analysis

**Fig. 1:**
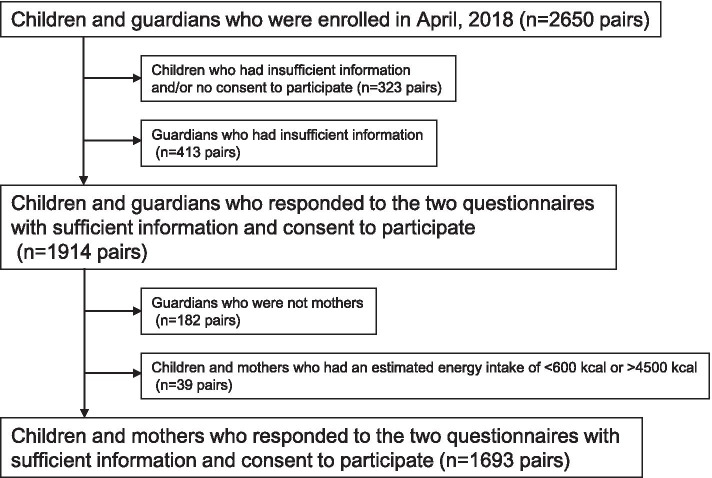
Flow diagram of participant selection for analysis

Among 2650 enrolled pairs of children and guardians, pairs that did not provide consent to participate or did not receive sufficient information through the questionnaire were excluded (Fig. 1). In addition, pairs in which the responding guardian was not the mother and those in which the energy intake of at least one of the pair, estimated using the BDHQ (BDHQ15y for child), was not between 600 and 4500 kcal were also excluded. Finally, 1693 mother and child pairs were included in the analysis (participation rate: 1693/2650*100 = 63.9%).

Food intakes of the children and mothers were compared by mothers’ employment status (not working, working < 8 h, and working ≥ 8 h) using a univariate linear regression model which included the children’s or mothers’ food intake as a dependent variable and mothers’ employment status as an independent variable. Trends of association were examined by assigning scores to the level of the independent variable (not working = 0, working <8h = 1, working ≥8h = 2). Then, the association between the selected food intake among children and mothers’ employment status was examined using multivariate linear regression models which included the children’s food intake as a dependent variable and mothers’ employment status as an independent variable. For these analyses, intakes of five foods were selected as outcomes, namely white rice, soy products, and vegetables, based on the results of univariate linear regression analysis in the children; and confectioneries and sweetened beverages as indicators of unhealthy dietary habits [17, 18]. Intakes of potatoes and bread were not analyzed irrespective of the results of univariate analysis because the amount of intake was relatively small and the influence of these two items on health is unclear. Further, based on the results for the relationship between food intakes and maternal employment status, the association between children’s BMI and maternal employment status was assessed.

The lifestyle factors listed below were considered possible confounders of the relationship between children’s food intake and mothers’ employment status. The multivariate models were adjusted using covariates for the child (sex, grade, nutrition knowledge, and attitude toward diet), mother (nutrition knowledge, attitude toward diet, food intake corresponding to the child [i.e., if children’s vegetable intake was a dependent variable in the model, mothers’ vegetable intake was used as a covariate], and sleeping hours on weekdays as an index of spare time), and family environment (cohabitants, socioeconomic status, frequency of eating out [as a counter index of home cooking], and frequency of communication between the child and mother). For white rice and confectioneries, trends of association were examined in the same manner as in univariate analysis. All analyses were performed with Stata/SE 15.1 for Windows (StataCorp LLC, Texas, USA). Statistical tests were two-sided, and *p* values of <0.05 were considered statistically significant.

## Results

The children and their mothers are characterized in Table 1. Among the mothers, 314 (18.5%) did not work, 798 (47.1%) worked < 8 h per day, and 581 (34.3%) worked ≥ 8 h per day.

**Table 1:** Characteristics of subjects (1693 pairs)

Variable	Category	*n* (%) or mean, SD		
		Children (*n*=1693)		Mothers (*n*=1693)
		Boys (*n*=821)	Girls (*n*=872)	
Personal factors				
Grade	5th	396 (48.2)	420 (48.2)	
	6th	425 (51.8)	452 (51.8)	
Age	Year	10.7, 0.6	10.6, 0.6	41.4, 4.9
Height	cm	142.2, 7.2	143.5, 7.4	158.3, 5.1
Weight	kg	36.6, 8.6	36.7, 8.2	54.1, 8.5
BMI	kg/m^2^	17.9, 3.1	17.7, 2.7	21.6, 3.2
Energy intake	kcal	2189, 673	1955, 642	1568, 455
Nutrition knowledge^a^	%	69.5, 13.6	70.2, 12.2	70.6, 14.8
Attitude toward diet	Inadequate	368 (44.8)	352 (40.4)	841 (49.7)
	Adequate	453 (55.2)	520 (59.6)	852 (50.3)
Maternal employment status	Not working	161 (19.6)	153 (17.6)	314 (18.5)
	Working (<8h)	376 (45.8)	422 (48.4)	798 (47.1)
	Working (≥8h)	284 (34.6)	297 (34.1)	581 (34.3)
Sleeping hours (weekdays)	Hour			6.3, 1.0
Family factors				
Cohabitation with husband^b^	No			183 (10.8)
(Child’s father)	Yes			1510 (89.2)
Cohabitation with mother^b^	No			1464 (86.5)
(Child’s grandmother)	Yes			229 (13.5)
Socioeconomic status	Straitened			537 (31.7)
	Average			946 (55.9)
	Affluent			210 (12.4)
Frequency of eating out^c^	< once a month	392 (47.8)	396 (45.4)	
	≥ once a month	429 (52.3)	476 (54.6)	
Frequency of communication	Low	510 (62.1)	508 (58.3)	504 (29.8)
about diet^d^	High	311 (37.9)	364 (41.7)	1189 (70.2)

*BMI* body mass index, *SD* standard deviation

^a^ 'Nutrition knowledge' is the percentage of correct answers to the nutrition knowledge questionnaire

^b^ Mother’s answer to questions about cohabitants. Mother's husband was the child's father, and mother's mother was the child's grandmother

^c^ Children’s answer on the frequency of eating out for dinner

^d^ Children’s answer on the frequency of communication with their guardian, and mother’s answered that with their child

Food intakes of the children and mothers are summarized by maternal employment status in Table 2.

**Table 2:** Children’s and mothers’ food intake and BMI by maternal employment status (*n*=1693)

Food group	Intake (g/1000kcal) or BMI (kg/m2) mean, SD			*P* value^a^
	Not working (*n*=314)	Working		
		<8h (*n*=798)	≥8h (*n*=581)	
Children				
BMI	17.6, 2.8	17.6, 2.8	18.2, 3.1	<0.01
White rice	174, 76	174, 77	185, 78	0.02
Bread	18, 14	18, 14	16, 14	0.04
Noodles	29, 17	30, 19	30, 19	0.50
Potatoes	16, 11	16, 11	14, 10	0.01
Soybean products	24, 19	22, 17	21, 15	0.02
Vegetables	102, 60	93, 57	91, 59	0.00
Fruits	26, 25	25, 28	27, 26	0.31
Fish and shellfish	29, 18	28, 17	28, 18	0.30
Meats	37, 19	37, 19	35, 18	0.17
Eggs	16, 11	16, 11	16, 11	0.40
Dairy products	109, 78	112, 85	113, 89	0.97
Confectioneries	49, 29	48, 26	47, 27	0.52
Sweetened beverage	64, 70	68, 75	70, 77	0.19
Mothers				
BMI	21.8, 3.7	21.5, 2.9	21.6, 3.3	0.99
White rice	158, 70	160, 67	159, 68	0.60
Bread	19, 14	22, 15	20, 15	0.94
Noodles	34, 21	35, 21	36, 25	0.69
Potatoes	23, 18	22, 17	22, 18	0.08
Soybean products	44, 28	37, 25	36, 26	<0.01
Vegetables	155, 75	143, 73	140, 78	<0.01
Fruits	32, 30	26, 25	27, 32	<0.01
Fish and shellfish	40, 21	35, 19	34, 20	<0.01
Meats	46, 19	46, 18	46, 20	0.93
Eggs	24, 15	24, 14	23, 16	0.24
Dairy products	63, 50	62, 55	65, 57	0.86
Confectioneries	38, 23	39, 23	41, 27	0.56
Sweetened beverage	24, 51	25, 42	28, 55	0.51

*BMI* body mass index

^a^ Trends of association were examined using a linear regression model which assigned scores to the level of the independent variable (not working = 0, working <8hr = 1, working ≥8hr = 2)

Longer working hours were associated with higher intake of white rice in children, and lower intakes of bread, potatoes, soybean products, and vegetables. For the mothers, longer working hours were significantly associated with their own lower intakes of soybean products, vegetables, fruits, and fish and shellfish, as well as higher intake of bread.

The results of multivariate linear regression analysis are shown in Table 3.

**Table 3:** Association of lifestyle factors with children’s food intake (n=1693)

Variable	Category (reference)	Children’s food intake (g/1000kcal)				
		White rice	Soybean products	Vegetables	Confectioneries	Sweetened beverage
		*β* 95%CI	*β* 95%CI	*β* 95%CI	*β* 95%CI	*β* 95%CI
Maternal employment status	Working <8h (vs not working)	1.9 [−7.9, 11.8]	−0.4 [−2.4, 1.7]	−4.1 [−11.1, 2.9]	−2.7 [−6.1, 0.7]	−0.9 [−10.5, 8.7]
	Working ≥8h (vs not working)	11.4 [1.0, 21.9] *	−1.4 [−3.5, 0.8]	−3.5 [−10.9, 3.9]	−4.0 [−7.6, −0.4] *	−0.8 [−10.9, 9.4]
Covariates for the child						
Sex	Girl (vs boy)	−26.3 [−33.4, −19.2] *	1.6 [ 0.1, 3.1] *	11.4 [ 6.4, 16.4] *	6.7 [ 4.2, 9.1] *	−2.2 [−9.1, 4.7]
Grade	6th (vs 5th)	−1.3 [−8.5, 5.9]	−0.2 [−1.7, 1.3]	2.9 [−2.2, 8.0]	−1.5 [−3.9, 1.0]	8.9 [2.0, 15.9] *
Nutrition knowledge^a^	Every 10% increase	−1.5 [−4.5, 1.5]	1.0 [ 0.4, 1.7] *	3.2 [1.0, 5.3] *	−1.6 [−2.7, −0.6] *	−4.1 [−7.0, −1.2] *
Attitude toward diet^b^	Adequate (vs inadequate)	2.5 [−4.9, 10.0]	−0.1 [−1.6, 1.5]	13.4 [8.1, 18.6] *	−2.5 [−5.1, 0.0]	−10.9 [−18.1, −3.6] *
Covariates for the mother						
Nutrition knowledge^a^	Every 10% increase	1.9 [−0.7, 4.4]	0.0 [−0.5, 0.5]	0.9 [−0.9, 2.7]	−0.8 [−1.7, 0.0]	−1.1 [−3.6, 1.4]
Attitude toward diet^b^	Adequate (vs inadequate)	−0.9 [−8.3, 6.4]	0.5 [−1.1, 2.0]	3.9 [−1.4, 9.1]	−1.2 [−3.7, 1.3]	−0.4 [−7.6, 6.8]
Corresponding food intake^c^	g/1000kcal	0.2 [0.2, 0.3] *	0.2 [0.2, 0.3] *	0.3 [0.2, 0.3] *	0.3 [ 0.2, 0.3] *	0.2 [0.2, 0.3] *
Sleeping hours (weekdays)	Hours	1.1 [−2.3, 4.6]	0.2 [−0.5, 0.9]	1.1 [−1.4, 3.5]	−0.6 [−1.8, 0.6]	−0.7 [−4.1, 2.6]
Family environment						
Cohabitation with husband (child’s father)	Yes (vs no)	−1.5 [−13.3, 10.3]	0.2 [−2.3, 2.6]	13.2 [4.9, 21.6] *	−0.6 [−4.6, 3.5]	−17.8 [−29.3, −6.3] *
Cohabitation with mother (child’s grandmother)	Yes (vs no)	0.8 [−9.9, 11.6]	−2.2 [−4.4, 0.0]	5.6 [−2.0, 13.2]	3.2 [−0.5, 6.8]	3.5 [−6.9, 13.9]
Socioeconomic status	Average (vs straitened)	3.8 [−4.3, 11.9]	0.9 [−0.8, 2.6]	4.8 [−1.0, 10.5]	1.4 [−1.4, 4.2]	−3.7 [−11.7, 4.2]
	Affluent (vs straitened)	24.3 [12.0, 36.6] *	−0.9 [−3.5, 1.6]	−7.7 [−16.4, 1.0]	3.4 [−0.8, 7.7]	−11.9 [−23.9, 0.1]
Frequency of eating out	≥ once a month (vs < once a month)	−3.8 [−7.9, 0.2]	−0.1 [−0.9, 0.7]	−3.6 [−6.4, -0. 7] *	2.6 [1.2, 4.0] *	2.2 [−1.8, 6.1]
Frequency of communication about diet (child: C, mother: M)	C:low-M:high (vs C:low-M:low)	3.2 [−6.6, 13.1]	0.1 [−1.9, 2.1]	−0.7 [−7.7, 6.3]	−0.1 [−3.5, 3.3]	−12.1 [−21.6, −2.5] *
	C:high-M:low (vs C:low-M:low)	7.5 [−6.6, 21.7]	−0.9 [−3.9, 2.0]	−3.0 [−13.0, 7.0]	−1.0 [−5.9, 3.9]	−6.0 [−19.8, 7.7]
	C:high-M:high (vs C:low-M:low)	6.3 [−4.3, 17.0]	0.8 [−1.4, 3.1]	7.5 [−0.1, 15.1]	−2.9 [−6.5, 0.8]	−12.7 [−23.1, −2.3] *

*CI* confidence interval

^a^“Nutrition knowledge” is the percentage of correct answers to the nutrition knowledge questionnaire

^b^Attitude toward diet was divided into adequate and inadequate based on responses to the questionnaire

^c^Mother’s corresponding food intake means the same kind of food intake as the child’s. For example, when the outcome was child’s vegetable intake, mother’s corresponding food intake was the mother’s vegetable intake

^*^P < 0.05

After adjustment for possible confounding factors, maternal employment status was significantly associated with higher intake of white rice (g/1000kcal) (*β* 11.4, 95% confidence interval (CI) [1.0, 21.9]; working ≥8h vs. not working) and lower intake of confectioneries (g/1000kcal) (*β* −4.0, 95%CI [−7.6, −0.4]; working ≥8h vs. not working) in the children. Trend of association was also significant between white rice/confectionery intake and three categories of maternal employment status (white rice, *β* 6.3, 95%CI [1.2, 11.4]; confectioneries, *β* −1.9, 95%CI [−3.7, −0.2]). In other words, factors other than maternal employment were significantly and consistently associated with food intakes in the children. The girls consumed more soybean products, vegetables, and confectioneries than the boys, but less white rice. Higher nutrition knowledge of the children was significantly associated with higher intakes of soybean products and vegetables and lower intakes of confectioneries and sweetened beverages. Adequate (better) attitude toward diet among the children was also associated with higher intake of vegetables and lower intake of sweetened beverage. Among mothers’ factors, corresponding food intakes among the mothers were consistently associated with their children’s food intake. Regarding family environment, cohabitation with the child’s father (the mother’s husband) was significantly associated with higher intake of vegetables and lower intake of sweetened beverage. Further, frequent eating out for dinner was associated with lower intake of vegetables and higher intake of confectioneries. Frequent communication about diet was associated with lower intake of sweetened beverage.

Since white rice is the main source of carbohydrate among Japanese, we also assessed the relationship between BMI in the children and maternal employment status (Table 4).

**Table 4:** Association of lifestyle factors with children’s BMI (*n*=1693)

Variable	Category (reference)	Crude	Adjusted
		*β 95%CI*	*β 95%CI*
Maternal employment status	Working <8h (vs not working)	0.05 [−0.3, 0.4]	0.06 [−0.3, 0.4]
	Working ≥8h (vs not working)	0.62 [0.2, 1.0] *	0.62 [ 0.2, 1.0] *
Covariates for the child			
Sex	Girl (vs boy)		−0.24 [−0.5, 0.0]
Grade	6th (vs 5th)		0.73 [0.5, 1.0] *
Nutrition knowledge^a^	Every 10% increase		−0.01 [−0.1, 0.1]
Attitude toward diet^b^	Adequate (vs inadequate)		0.19 [−0.1, 0.5]
Covariates for the mother			
Nutrition knowledge^a^	Every 10% increase		−0.05 [−0.1, 0.0]
Attitude toward diet^b^	Adequate (vs inadequate)		0.17 [−0.1, 0.4]
BMI	kg/m^2^		0.24 [0.2, 0.3] *
Sleeping hours (weekdays)	Hours		0.09 [0.0, 0.2] *
Family environment			
Cohabitation with husband (child’s father)	Yes (vs no)		−0.20 [−0.6, 0.3]
Cohabitation with mother (child’s grandmother)	Yes (vs no)		−0.08 [−0.5, 0.3]
Socioeconomic status	Average (vs straitened)		−0.20 [−0.5, 0.1]
	Affluent (vs straitened)		−0.60 [−1.1, −0.1] *
Frequency of eating out	≥ Once a month (vs < once a month)		−0.14 [−0.3, 0.0]
Frequency of communication about diet (child:C, mother:M)	C:low-M:high (vs C:low-M:low)		0.11 [−0.3, 0.5]
	C:high-M:low (vs C:low-M:low)		0.19 [−0.3, 0.7]
	C:high-M:high (vs C:low-M:low)		0.10 [−0.3, 0.5]

*BMI* body mass index, *CI* confidence interval

^a^“Nutrition knowledge” is the percentage of correct answers to the nutrition knowledge questionnaire

^b^Attitude toward diet was divided into adequate and inadequate based on responses to the questionnaire

^*^P < 0.05

Even after adjusting for covariates in the multivariate model, longer working hours in mothers was associated with higher BMI in children (kg/m^2^) (*β* 0.62, 95%CI [0.2, 1.0]; working ≥8h vs. not working). A higher BMI of mothers was also associated with a higher BMI of the children.

## Discussion

To our knowledge, this is the first study to investigate the relationship between children’s food intake and maternal employment status in Japan. When mothers had jobs and their working hours were longer, the white rice intake of their children was higher and the intake of confectioneries was lower. In addition, longer maternal working hours was significantly associated with higher BMI in the children. In contrast to previous studies, we found that maternal employment status was not associated with unhealthy food choices, including no decrease in vegetable intake [3, 4, 6] and no increase in sweetened beverage intake [4]. Nevertheless, children’s energy intake was likely affected through the increase in white rice intake.

White rice (refined rice) is a major staple food for Japanese, and it is easily speculated that the preparation of white rice is simple and time-saving for busy mothers due to the wide use of electric rice cookers in Japan (household penetration, 89%) [19]. Although obesity rates among primary school students in Japan are not particularly high (10.0% in boys and 8.8% in girls aged 11 years in 2018) [20], the intake of white rice among children with working mothers warrants attention. The same association between higher BMI and maternal employment has been reported previously [4, 21–24]. Also, a higher intake of white rice has been associated with risk of type 2 diabetes in adults [25]. The health effect of white rice intake in childhood warrants further investigation.

In contrast, our present study found that the intakes of other foods such as vegetables/soybean products (usually included in side dishes) or fish/meats (usually included in main dishes) in children did not differ by maternal employment status. These results differ from previous studies which reported that longer maternal working hours had negative effects on children’s dietary habits [3, 5]. In particular, although the intake of confectioneries is usually considered a marker of broader unhealthy dietary habits [26], we found that intake was lower in children whose mothers worked for longer hours. Since nearly 90% of the BDHQ15y was completed by the children themselves or the children and the guardian together, irrespective of maternal employment status, the possibility of underreporting of snack intake by the working mothers was unlikely.

The difference between these present and past results might be due to the covariates included in the respective models. Several past studies adopted race, mother’s educational background, mother’s childbirth age, socioeconomic-status, and household income as covariates [3–5]. In contrast, our present models included nutrition knowledge, attitude toward diet, mothers’ food intake, family structure, and communication between child and mother, which were not assessed in these previous studies. Although these factors might be associated with maternal and family background factors, such as education, household income or occupation, some were more closely associated with food intake in the children than maternal employment status. The association between higher nutrition knowledge and higher intake of vegetables among children was consistent with previous studies [16, 27]. For soybean products, a similar relationship has been reported for girls [16]. A better understanding of nutrition and foods and the acquisition of suitable attitudes towards diet are thought to be important to the achievement of good dietary habits in children.

Regarding maternal covariates, correspondence between the mothers’ food intake and that of their children was the only factor which was significantly associated with the children’s food intake. Previous studies reported that the amount of food mothers served themselves was associated with the amount they served their children [28] and that higher vegetable intake of mothers was associated with higher vegetable intake among their children [29]. We observed a similar association between the food intake of mothers and children. This finding is considered well plausible, given that the mothers both answered the questionnaires and were the main preparers of meals for the children and—apart from school lunches—consumed most of the meals with their children. In contrast, the mothers’ nutrition knowledge and attitude toward diet were not associated with the intake of any food in the children. Behavior—that is, the actual selection of dishes and foods by the mothers—was thought to be more important to improving dietary habits among the children than the mother’s nutrition knowledge and attitude toward diet. Similar results were seen in another study from Japan, which reported that nutrient intake among junior high school students did not differ no matter whether the mother had high or low nutrition knowledge [30]. In contrast, other studies reported different results [31, 32], indicating the need for further investigation of the influence of mothers’ nutrition knowledge and attitude toward diet on children’s dietary intake. The effect may differ by children’s age.

Among family environment factors, cohabitation with the child’s father and frequency of eating out for dinner were significantly associated with the intakes of two foods. Average annual income of single-parent families, particularly mother-child families (3.48 million Japanese yen in 2015 [33]), was lower than that of all families which included children (7.08 million Japanese yen in 2015 [2]). Cohabitation with the child’s father might have been an index of the financial condition of the family. Moreover, cohabitation and the frequency of eating out may be indexes of the mother’s spare time. A survey by The Japan Institute for Labour Policy and Training in 2014 [34] reported that the proportion of mothers who thought that working time was too long to allow sufficient time for housework and child care was higher in mother-child families than in two-parent families (58.4% vs. 48.2%). Establishment of a better food environment appears to be an important means of supporting busy families, particularly those with time and income limitations. For example, facilitated or subsidized access to time-saving processed foods, such as cut vegetables, frozen vegetables, and semi-prepared foods would likely facilitate children and their guardians in consuming healthier meals. Communication about diet between children and mothers was another factor associated with sweetened beverage intake. Frequent communication, particularly as recognized by the mothers, was associated with a decrease in this intake. More communication between mothers and their children about diet is a potential means of promoting better dietary habits among children.

Our study has several strengths. First, the survey participants were recruited from all five administrative districts in the prefecture, which was considered to be highly representative of the region. The participation rate was relatively high (1693/2650 * 100 = 63.9%). Second, the children and their mothers were surveyed simultaneously, and their data were combined to assess their relationship. Third, food intakes were quantitatively assessed by the BDHQ and the BDHQ15y, both of which have been validated [12, 13]. Fourth, the statistical model made sufficient adjustment for confounding factors. For example, nutrition knowledge, attitude toward diet, and communication between children and mothers were newly assessed with maternal employment status.

Several limitations of the study should be mentioned. First, given that the study was conducted in a single prefecture in Japan, the generalizability of the results should be carefully considered. Nevertheless, the prefecture is located in the central part of the main island of Japan, includes both urban and rural areas, and is middle ranked among Japan’s 47 prefectures with regard to area, population, and population density. In addition, maternal employment in this prefecture was similar to that in a national survey [35]. The age range of the participating children was limited to 10 to 12 years, and this inclusion criterion also hampered the generalizability of the study. Although it is difficult to collect information from children younger than those in the present study, conducting a similar study in toddlers or preschool children should be useful. Second, maternal employment was evaluated in terms of working hours per day only, and other factors such as night shifts, self-employment, number of working days per week, and commuting time were not considered. These other aspects of working style should be considered in future studies. Third, children’s attitudes toward diet were ascertained using a single item only, which has not been validated. Nevertheless, given that children’s attitudes toward diet were significantly associated with food intake, the question used in the present study (“Do you pay attention to whether your diet is healthy or not?”) likely captured certain aspects of attitude toward diet.

## Conclusion

This study showed that longer maternal working hours were significantly associated with higher intake of white rice and lower intake of confectioneries, as well as higher BMI among children. In contrast, other factors such as children’s nutrition knowledge, children’s attitudes toward diet, mothers’ food intake, and family environment factors affected the intake of the majority of foods among the children, including that of vegetables and sweetened beverages. Even when a mother works, it may be possible to improve her children’s dietary intake by other means, such as nutrition education for children or enhancement of the food environment.

## Data Availability

The datasets used and/or analyzed during the current study are available for research purpose from the corresponding author on reasonable request.

## References

[1] Yamamoto J, Oura Y, Tamaki S, Yagi K (2017). Effects of employment status of women and presence of children on dependency on processed foods (in Japanese). Japan J Farm Manag.

[2] Ministry of Health Labour and Welfare: Comprehensive Survey of Living Conditions. 2018. https://www.mhlw.go.jp/toukei/saikin/hw/k-tyosa/k-tyosa18/index.html. Accessed Mar.

[3] Wu JC (2018). Parental work characteristics and diet quality among pre-school children in dual-parent households: results from a population-based cohort in Taiwan. Public Health Nutr..

[4] Datar A, Nicosia N, Shier V (2014). Maternal work and children’s diet, activity, and obesity. Soc Sci Med..

[5] Hawkins SS, Cole TJ (2009). Law C, Millennium Cohort Study Child Health G. Examining the relationship between maternal employment and health behaviours in 5-year-old British children. J Epidemiol Community Health..

[6] Bauer KW, Hearst MO, Escoto K, Berge JM, Neumark-Sztainer D (2012). Parental employment and work-family stress: associations with family food environments. Soc Sci Med..

[7] Li J, O'Sullivan T, Johnson S, Stanley F, Oddy W (2012). Maternal work hours in early to middle childhood link to later adolescent diet quality. Public Health Nutr..

[8] Wadhera D, Capaldi Phillips ED, Wilkie LM, Boggess MM (2015). Perceived recollection of frequent exposure to foods in childhood is associated with adulthood liking. Appetite..

[9] Aatola H, Koivistoinen T, Hutri-Kahonen N, Juonala M, Mikkila V, Lehtimaki T (2010). Lifetime fruit and vegetable consumption and arterial pulse wave velocity in adulthood: the Cardiovascular Risk in Young Finns Study. Circulation..

[10] Kaikkonen JE, Mikkila V, Raitakari OT (2014). Role of childhood food patterns on adult cardiovascular disease risk. Curr Atheroscler Rep..

[11] Ministry of Health Labour and Welfare: Article 32 of the Labor Standards Act. https://www.mhlw.go.jp/web/t_doc?dataId=73022000&dataType=0&pageNo=1. Accessed March.

[12] Kobayashi S, Murakami K, Sasaki S, Okubo H, Hirota N, Notsu A (2011). Comparison of relative validity of food group intakes estimated by comprehensive and brief-type self-administered diet history questionnaires against 16 d dietary records in Japanese adults. Public Health Nutr..

[13] Kobayashi S, Honda S, Murakami K, Sasaki S, Okubo H, Hirota N (2012). Both comprehensive and brief self-administered diet history questionnaires satisfactorily rank nutrient intakes in Japanese adults. J Epidemiol..

[14] Walter W (2013). Nutritional Epidemiology.

[15] Murakami K, Sasaki S, Uenishi K (2012). The degree of misreporting of the energy-adjusted intake of protein, potassium, and sodium does not differ among under-, acceptable, and over-reporters of energy intake. Nutr Res..

[16] Asakura K, Todoriki H, Sasaki S (2017). Relationship between nutrition knowledge and dietary intake among primary school children in Japan: combined effect of children’s and their guardians’ knowledge. J Epidemiol..

[17] Ludwig DS, Peterson KE, Gortmaker SL (2001). Relation between consumption of sugar-sweetened drinks and childhood obesity: a prospective, observational analysis. Lancet..

[18] Nissinen K, Mikkilä V, Männistö S, Lahti-Koski M, Räsänen L, Viikari J (2009). Sweets and sugar-sweetened soft drink intake in childhood in relation to adult BMI and overweight. The Cardiovascular Risk in Young Finns Study. Public Health Nutr..

[19] Ministry of Internal Affairs and Communications: National Survey of Consumption. https://www.stat.go.jp/data/zensho/2014/kekka.html (2014). Accessed March.

[20] Ministry of Education Culture Sports Science and Technology: School Health Examination Survey. https://www.mext.go.jp/b_menu/toukei/chousa05/hoken/kekka/k_detail/1411711.htm (2018). Accessed March.

[21] Gaina A, Sekine M, Chandola T, Marmot M, Kagamimori S (2009). Mother employment status and nutritional patterns in Japanese junior high schoolchildren. Int J Obes (Lond)..

[22] Watanabe E, Lee JS, Kawakubo K (2011). Associations of maternal employment and three-generation families with pre-school children’s overweight and obesity in Japan. Int J Obes (Lond)..

[23] Ziol-Guest KM (2014). A commentary on “maternal work and children’s diet, activity, and obesity”. Soc Sci Med..

[24] Koca T, Akcam M, Serdaroglu F, Dereci S (2017). Breakfast habits, dairy product consumption, physical activity, and their associations with body mass index in children aged 6-18. Eur J Pediatr..

[25] van Dam RM (2020). A global perspective on white rice consumption and risk of type 2 diabetes. Diabetes Care..

[26] Vik FN, Heslien KEP, Van Lippevelde W, Øverby NC (2020). Effect of a free healthy school meal on fruit, vegetables and unhealthy snacks intake in Norwegian 10- to 12-year-old children. BMC Public Health..

[27] Wardle J, Parmenter K, Waller J (2000). Nutrition knowledge and food intake. Appetite..

[28] Johnson SL, Hughes SO, Cui X, Li X, Allison DB, Liu Y (2014). Portion sizes for children are predicted by parental characteristics and the amounts parents serve themselves. Am J Clin Nutr..

[29] Tada Y, Tomata Y, Sunami A, Yokoyama Y, Hida A, Furusho T (2015). Examining the relationship between vegetable intake of mothers and that of their children: a cross-sectional study of 10- to 12-year-old schoolchildren in Japan. Public Health Nutr..

[30] Matsumoto M, Hatamoto Y, Masumoto A, Sakamoto A, Ikemoto S. Mothers’ nutrition knowledge is unlikely to be related to adolescents’ habitual nutrient intake inadequacy in Japan: a cross-sectional study of Japanese junior high school students. Nutrients. 2020;12. 10.3390/nu12092801. 10.3390/nu12092801 PMC7551575 32933110

[31] Yung TKC, Lee A, Ho MM, Keung VMW, Lee JCK (2010). Maternal influences on fruit and vegetable consumption of schoolchildren: case study in Hong Kong. Matern Child Nutr..

[32] Al-Shookri A, Al-Shukaily L, Hassan F, Al-Sheraji S, Al-Tobi S (2011). Effect of mothers nutritional knowledge and attitudes on omani children’s dietary intake. Oman Med J..

[33] Ministry of Health Labour and Welfare: Nationwide Survey on Single parent household. https://www.mhlw.go.jp/stf/seisakunitsuite/bunya/0000188147.html (2016). Accessed March.

[34] The Japan Institute for Labour Policy and Training: Survey on Living Conditions of Households with Children and Employment of Their Parents 2014 (3rd National Survey of Households with Children). http://www.jil.go.jp/institute/research/2015/145.html (2014). Accessed March.

[35] Ministry of Health Labour and Welfare: National Health and Nutrition Survey. . https://www.mhlw.go.jp/stf/seisakunitsuite/bunya/kenkou_iryou/kenkou/eiyou/h29-houkoku.html (2017). Accessed March.

